# The Epidemiology of Sudden Oak Death Disease Caused by *Phytophthora ramorum* in a Mixed Bay Laurel-Oak Woodland Provides Important Clues for Disease Management

**DOI:** 10.3390/pathogens11020250

**Published:** 2022-02-15

**Authors:** Melina Kozanitas, Margaret R. Metz, Todd W. Osmundson, Maria Socorro Serrano, Matteo Garbelotto

**Affiliations:** 1 Department of Environmental Science, Policy & Management, University of California, 137 Mulford Hall, Berkeley, CA 94702, USA; mkozanitas@gmail.com (M.K.); mariazerrano2@gmail.com (M.S.S.); 2 Department of Biology, Lewis & Clark, 615 S. Palatine Hill Rd., Portland, OR 97219, USA; mmetz@lclark.edu; 3 Department of Biology, University of Wisconsin-La Crosse, 3034 Cowley Hall, La Crosse, WI 54601, USA; tosmundson@uwlax.edu

**Keywords:** biological invasions, disease ecology, *Quercus agrifolia*, refugial host, superspreader, *Umbellularia californica*

## Abstract

Epidemiological models are important for the understanding of disease progression in plants and for the design of control strategies. *Phytophthora ramorum*, the pathogen responsible for the disease known as Sudden Oak Death, causes lethal infection on several oaks but relies on California bay laurels for transmission. Here, repeated surveys of bay laurels and oaks indicated that bay laurel disease incidence was positively correlated with rainfall, bay laurel density, and an eastern aspect, and negatively correlated with bay laurel basal area. Oak infection only occurred in years when rainfall was higher than the 30-year average, and although infection rates were greater among larger trees, mortality was greater among smaller trees. Additionally, larger oaks closer to infected bay laurels exhibited greater infection rates. Disease incidence differed among sites, and only a fraction of bay laurels were disease superspreaders, while even fewer individuals were refugial trees harboring active infections during dry periods. Based on this study, reducing bay laurel density in denser stands and the number of superspreaders or refugial trees in less dense stands may reduce disease incidence. However, the selective removal of bay laurel trees 0–10 m from oaks is likely to be more effective in preventing infection of specific oaks.

## 1. Introduction

The role of pathogen transmission and host competence heterogeneity at individual tree- and population-level scales is increasingly recognized as an important driver of plant disease epidemics [1,2,3,4]. Unlike hosts in most zoonotic disease systems, plants are sessile and, thus, the understanding of plant disease dynamics must rely more heavily on the spatial context of the system [5]. Particular individuals, but also the sites in which these particular individuals grow, may have an outsized effect on disease transmission, earning the designations of “superspreading” hosts and “hotspots,” respectively [6]. Factors that influence host susceptibility (e.g., genetics and the host’s microbiome or other symbiotic interactions) as well as environmental factors (e.g., climate, weather, slope, aspect, and host density) may allow a particular individual to function as an infection “superspreader” [7] or as an inoculum reservoir (“refugial” host) during unfavorable periods [8]. Because trees are sedentary, sites where superspreaders are present might serve as hotspots for disease spread and sites containing refugial trees could become starting points for future outbreaks due to a pathogen’s ability to survive in these inoculum reservoirs during climatically unfavorable periods.

Many emergent forest diseases, such as those caused by introduced fungi and oomycetes, have the ability to cause large-scale landscape transformations [9,10,11,12,13,14]. Control of these diseases on a broad scale is expensive and often impractical, and treatment of symptomatic hosts alone is unlikely to control disease epidemics [15]. Management interventions for forest diseases could be efficiently focused to limited geographic areas and timeframes if it were possible to identify those site or climatic variables that are correlated with higher infection levels. Likewise, knowing the location and the characteristics of sites where superspreading individuals and/or refugial hosts are likely to occur would help us to better understand the epidemiology of a disease and to identify effective control strategies. This is particularly true for a disease such as Sudden Oak Death (SOD), which is the topic of this study, characterized by a large, fragmented, range, and by the cryptic or quasi-cryptic infection status of many plant hosts [16]. 

Oomycetes are not fungi and belong to the Kingdom Stramenopila; however, they include a large number of emergent tree and plant pathogens [17]. Sudden Oak Death, caused by the introduced and invasive oomycete *Phytophthora ramorum*, is a multi-host disease that has caused widespread mortality of coast live oak (*Quercus agrifolia*) in California and tanoak (*Notholithocarpus densiflorus*) in Oregon and California [18]. *Phytophthora ramorum* can infect over 100 plant species [19], but there is great heterogeneity among hosts regarding their infectivity and susceptibility. While certain oak species and tanoaks are most susceptible to the lethal form of the disease, tanoaks and California bay laurels (*Umbellularia californica*) support significant levels of sporulation by the pathogen. Tanoaks are epidemiologically most relevant for the spread of SOD in redwood-tanoak or pure tanoak forest stands, while California bay laurel (or simply bay laurel) is the primary host responsible for pathogen transmission in California mixed oak woodlands [20,21,22]. *Phytophthora ramorum* produces relatively inconspicuous leaf lesions on bay laurels that can be regarded as cryptic or quasi-cryptic compared to the more visible symptoms on oaks [23]; however, infected bay laurel leaves support very abundant production of both infectious zoospores and survival structures (chlamydospores) [21]. *Phytophthora ramorum* does not kill bay laurels, at most causing early leaf drop of a limited number of infected leaves in warmer sites [24]. The pathogen exhibits strong seasonal behavior on bay laurel leaves, producing infectious spores during the wet spring season and surviving in either a viable (i.e., live culturable) or a non-viable (i.e., live and non-culturable) state during the dry summer months in California’s Mediterranean climate [13,22,25,26]. Coast live oaks are dead-end hosts for the disease, as they contract lethal infections but cannot effectively transmit the pathogen. Establishment of the pathogen on oaks generally results in large visible “bleeding” cankers on the trunk. Necrosis of the cambial tissue eventually leads to girdling and death of the tree [27]. 

High levels of disease incidence at the landscape level in mixed oak woodlands are most often correlated to stand dominance of bay laurel, the major transmissive host in the disease system [22,24,28]. Because of the epidemiological role bay laurels play in the spread of SOD, it is important not only to identify factors that promote colonization of bay laurel leaves by the pathogen, but also to understand sources of heterogeneity in pathogen transmission among bay laurels and from bay laurel to oaks and tanoaks. Previous studies of heterogeneity in this system have indicated that in vitro susceptibility of bay laurel leaves to *P. ramorum* infection correlates to bay laurel provenance and possibly genetics [29], but overall prevalence of infection in the field correlates more strongly with environmental characters [30]. Similarly, the ability of the pathogen to survive throughout the summer season is correlated with site-level characteristics [31]. Finally, reversal of infectious status of bays from infected to uninfected has been shown to be strongly correlated with topography, weather, and forest stand structure [32].

Despite the recent advances in our understanding of the biology of *P. ramorum* and of the different epidemiological roles provided by each of its various host species, the degree of heterogeneity in infection and transmissive potential among individual host plants within single bay laurel provenances or forest stands has not been extensively studied. For example, bay laurel trees that consistently support viable pathogen sporulation during the infection-favorable rainy season are likely to be more important as superspreaders, while trees harboring pathogen infection during the dry season may be more important as reservoirs or refugial hosts for the disease, allowing the pathogen to survive year to year through unfavorable conditions. The degree of linkage between this heterogeneity and the ultimate effect of the epidemic, measured in rates of oak and tanoak mortality, and the environmental or other factors that influence this linkage are not well understood.

This study examined bay laurel infection at the individual and plot levels and its association with oak infection and mortality across seasons and years in order to understand the role of individual, spatial, environmental, and temporal heterogeneity in propagating the SOD epidemic. Specifically, the following questions and associated hypotheses were addressed:

(1) Which characteristics of the biotic and abiotic neighborhood are most important in predicting infection of the transmissive host bay laurel?

**H1:**  *plot-level disease prevalence on bay laurel is correlated with rainfall and aridity, basal area, and density of bay trees.*

**H2:**  *tree-specific characteristics, including diameter at breast height (DBH), aspect, and canopy cover of individual trees, are correlated with levels of disease prevalence on bay.*

(2) Are oak infection and oak mortality correlated with oak size, high levels of bay laurel infection, and/or close proximity to infected bay laurels?

**H3:**  *oak infection is correlated with high levels of bay laurel disease prevalence, high bay laurel density, and close proximity to infected bay laurels.*

**H4:**  *oak infection and oak mortality are correlated with intrinsic characters of oak individuals, such as oak size; oak mortality is also correlated with the presence of visible SOD symptoms on the trunk (i.e., bleeding bole cankers).*

(3) Are there trees and sites that are epidemiologically more relevant?

**H5:**  *some sites will be hotspots containing a significant number of superspreaders and refugial trees.*

**H6:**  *hotspots will be characterized by overall higher cumulative disease incidence, as well as by higher disease incidence when climate is favorable to the pathogen’s transmission.*

**H7:**  *bay laurel density will be higher and basal area lower in hotspots than in other sites.*

The results of this study suggest some avenues for formulating efficient and cost-effective disease mitigation efforts. Information useful for this purpose includes knowledge of: (a) bay laurels that are more likely to infect oaks; (b) oaks that are more susceptible to infection and/or mortality; (c) forest stand characteristics that are correlated with higher disease incidence; and (d) presence of bay laurel individuals that may be either superspreaders of disease and/or refugial hosts for the pathogen. 

## 2. Results

**Figure 1:**
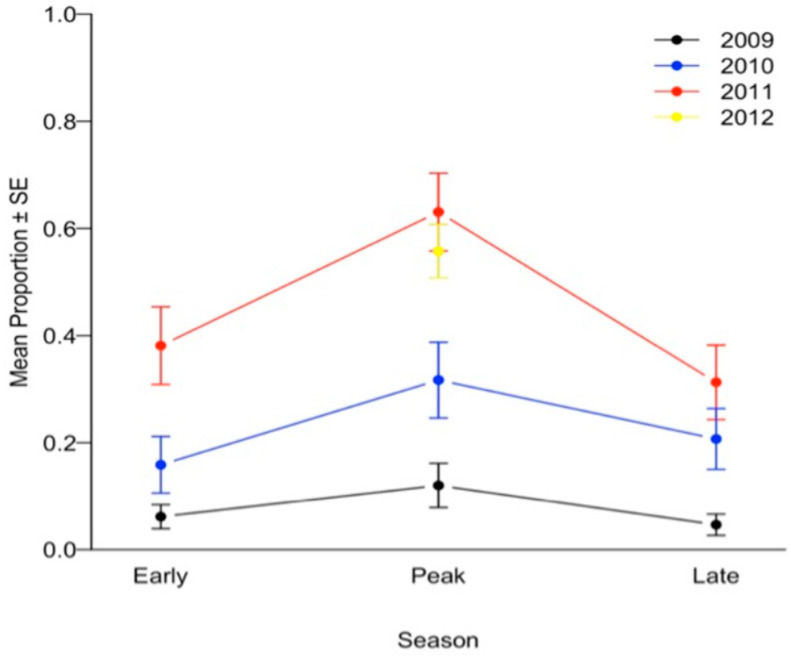
Isolation success of *Phytophthora ramorum* from bay laurel leaf samples collected over 10 sampling events, three times per year for three years from 2009–2011, and in the peak season of 2012. Early, peak, and late sampling events represent late winter, late spring, and autumn, respectively.

**Figure 2:**
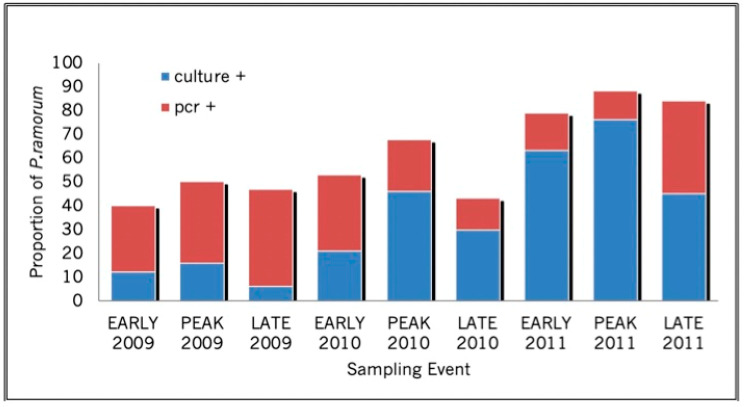
Annual and seasonal heterogeneity in proportions of bay laurel infection with viable (culture +) and non-viable (culture −/PCR +) *Phytophthora ramorum* over three years. Blue bars represent the proportion of bay laurel trees in all plots that yielded a positive *P. ramorum* isolate. Red bars represent the proportion of samples that were negative in culture, but positive when tested with a PCR diagnostic assay.

Pathogen viability on bay laurel leaves increased with rainfall. For the six plots for which rainfall and temperature data were available allowing us to calculate the AI (Table S3), the logistic regression of *P. ramorum* total incidence (Table S2) against AI values showed a significant and positive relationship between AI and prevalence of *P. ramorum* (chi-square = 16.2, *p* < 0.0001). Levels of active culturable infections were also highest in the peak sampling period of each year compared to the other two sampling times of the same year (Figure 1). Early season sampling periods yielded higher isolation success than late season sampling periods, with the exception of one late sampling period (autumn 2010) when unseasonably early rains occurred a week before the sampling [33]. Rainfall increased each year between 2009 and 2011 and declined slightly in 2012, and total isolation success also increased each year, and then dropped in the peak sampling period of 2012 (Figure 2). Peak 2012 was characterized by less rainfall than peak 2011, with rainfall values at 66 and 119% of the 30-year average, respectively. In late 2014, with rainfall levels down to about 50% of the 30-year average, overall bay laurel infection was lower than previously measured, further demonstrating that foliar infection cycles up and down, depending on rainfall, as previously shown by Kozanitas et al. [33].

Total infection levels, including both culture and PCR positives, exhibited an overall increase over the course of the study; however, with each subsequent sampling event, the proportion of non-viable infection decreased relative to active infection until the late 2011 sampling period, when non-viable infection and active infection levels were nearly equal (Figure 2). It should be noted that PCR positive-culture negative samples may include living (viable and non-viable) as well as dead infections; however, Chimento et al. [34] have estimated that 69% of PCR samples, identified as positive for *Phytophthora ramorum* using the same PCR assay employed here, were living.

**Figure 3:**
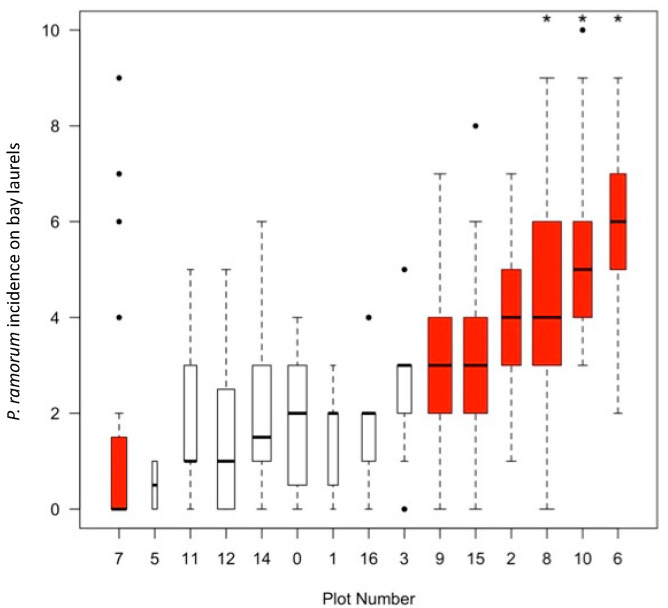
Comparison of *Phytophthora ramorum* disease incidence on bay laurel (*Umbellularia californica*) among plots across ten sampling events over four years. Plots are ranked in order from lowest to highest incidence. The bold line in each box represents the median number of times a tree in that plot yielded a positive culture, while the width of the box is proportional to the number of trees in a plot. Whiskers extend to 10th and 90th percentiles of bay laurel infection incidence, while dots are outliers beyond those specifications, but each dot may represent one or multiple datapoints. Red colored bars indicate the presence of disease superspreading trees, bay laurels that yielded a positive isolate of *P. ramorum* >7/10 times. Plots denoted with an asterisk contain one or more “refugial” trees that harbor viable infection in all three autumn (late season) sampling events, have the highest level of infection and the most disease superspreaders.

Active infection on bay laurel was found to be heterogeneous at both spatial and individual scales. Comparisons of cumulative disease incidence (CDI) across plots and levels of infection frequency among individuals, showed heterogeneity both among plots and among individual trees within plots (Figure 3). Disease hotspots, defined on the basis of containing at least four putative superspreader trees, were also more likely to contain refugial trees; in six of the seven hotspots, active infection was detected in refugial hosts in at least two of the three late seasons, and the three plots with the highest median CDI, each contained refugial trees that were positive in all three of the late seasons (Figure 3). 

**Figure 4:**
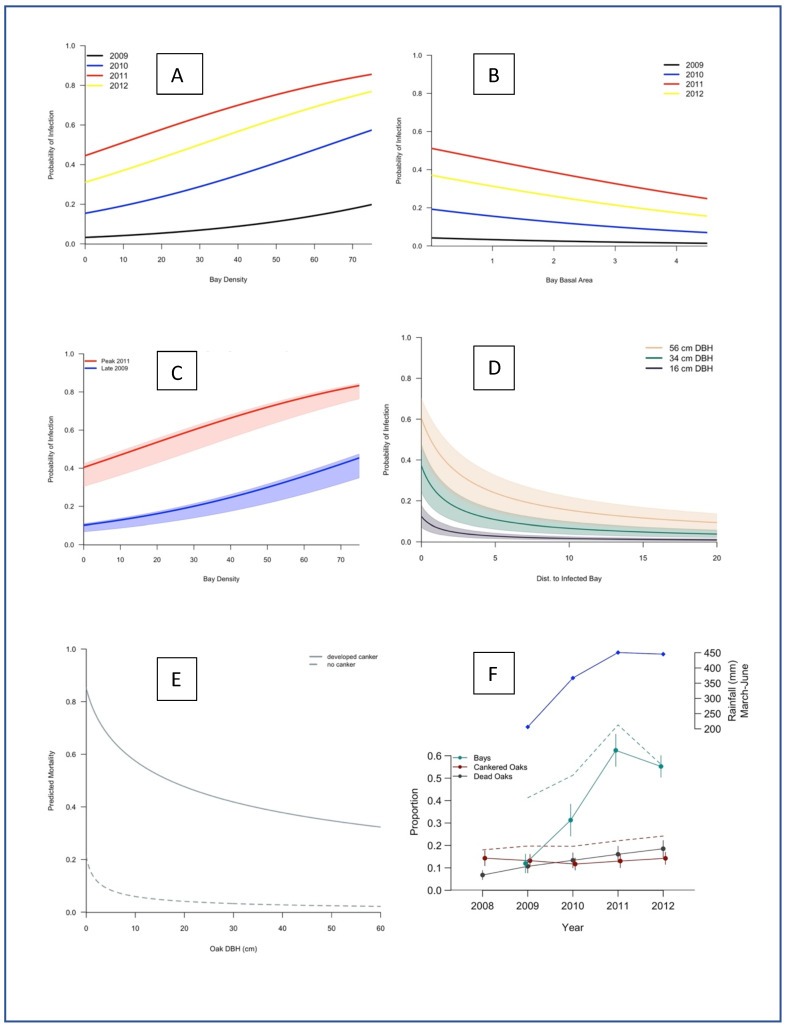
(**A**) Probability of active *Phytophthora ramorum* infection on bay laurel (*Umbellularia californica)* with respect to bay laurel density, with bay laurel basal area held constant, in each year of the study. (**B**) Probability of active *P. ramorum* infection on bay laurel in relation to bay laurel basal area, with bay laurel density held constant, in each year of the study. (**C**) Probability of active *P. ramorum* infection on an average-sized bay laurel in the wettest and driest conditions encountered during the study (driest: late 2009; wettest: peak 2011) with respect to bay density. Bold center line represents median bay laurel basal area, the “cloud” around the center lines represents the 90th and 10th percentiles of the data. (**D**) Probability of infection on coast live oak (*Quercus agrifolia*) as a function of size (diameter at breast height or DBH), bay laurel density, and distance to nearest infected bay laurel tree for three size classes of oaks. The cloud around each line represents bay laurel density. (**E**) Predicted oak mortality based on oak DBH and presence of symptomatic oak infection (canker). The solid line represents oaks symptomatic for *P. ramorum* infection, and the dashed line represents asymptomatic trees. (**F**) Association of rainfall with active *P. ramorum* infection on bay laurel, oak infection, and oak mortality in each year of the study. The blue line represents the amount of rainfall during the spring of each year. The solid green line shows the proportion of active infection on bay laurel from 2009 to 2012, and the dashed green line includes the proportion of non-viable bay laurel infection detected from PCR diagnostics. The red solid line represents the proportion of infected oak trees each year, the black solid line represents the proportion of dead oaks. The red dashed line combines the proportion of cankered oaks and dead oaks each year.

Bay laurel infection was also found to vary with bay density, topography, and year. Results of the first GLMM (Table 1) indicated that the probability of a bay laurel being infected by *P. ramorum* significantly increased with bay density (Figure 4A) and decreased as bay basal area increased (Figure 4B); i.e., being surrounded by a larger number of smaller trees led to higher probability of infection for any given bay laurel tree. This is true because, in general, higher basal area is found in stands with fewer larger trees. Infection was also more likely to occur on east-facing slopes than on slopes with a different aspect (Table 1). Likelihood of infection was significantly higher during the peak season than either the early or late season, and infection was more likely to occur in the early than in the late season (peak > early > late) (Table 1). The likelihood of bay laurels being infected was found to be significantly different each year (Table 1). Infection probability was highest in 2011, and lowest in 2009, mirroring the proportion of positive isolates (2011 > 2012 > 2010 > 2009) (Figure 1). The likelihood of a single average-sized bay laurel tree becoming infected with varying bay density and basal area under two opposing climatic conditions was determined by comparing results from the wettest (peak 2011) and driest (late 2009) seasons. In both instances, infection probability increased only with bay density (Figure 4C).

The next two models indicated that the likelihood of oak infection and oak survival varied significantly with oak size and different bay laurel neighborhood effects. Oak infection probability was positively correlated with oak size (DBH), bay laurel density, and proximity to the closest infected bay laurel foliage (Table 1). The proximity effect was strongest when oaks were between 0 and 5 m from infected bay laurel foliage, with the likelihood of infection decreasing to almost zero when that distance was greater than 10 m (Figure 4D). Oak mortality determined by the presence of brown foliage, brown cambium, bark beetle attacks, and *Annulohypoxylon thouarsianum* fruitbodies on the stem was significantly positively correlated with the presence of a canker and negatively correlated with oak DBH (Figure 4E). The association with oak DBH was opposite for oak infection. While a larger DBH was positively correlated with a higher probability of oak infection, a smaller DBH led to a higher likelihood of oak mortality (Figure 4E). 

**Figure 5:**
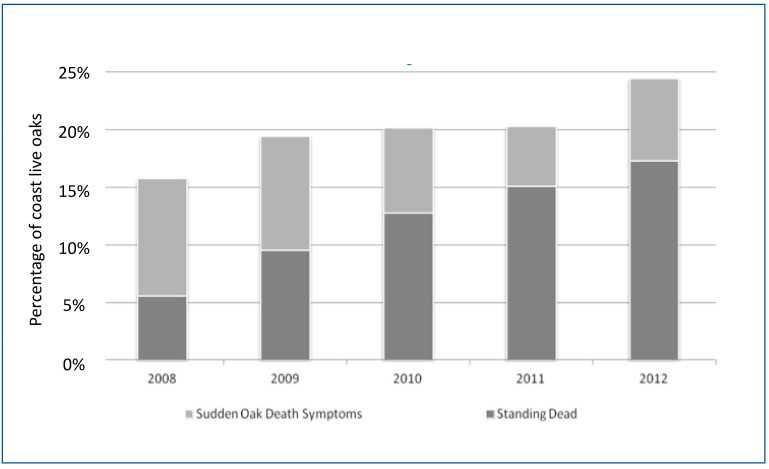
Yearly percentage of infection on coast live oak (*Quercus agrifolia*) (light grey) and mortality (dark grey) over the course of the study.

Oak mortality exhibited an overall increase over the course of the study, while the presence of new disease symptoms in oaks decreased between 2008 and 2011, and then increased in 2012 (Figure 5). Likewise, the number of cankered oaks progressively decreased during drier spells (2008–2010) due to mortality of infected oaks and lack of new infections. With the onset of significant rain events in 2011, new oak infections occurred across most of the plot network for the first time over the course of the study, and in 2012, these new infections became generally visible (Figure 4F and Figure 5; Table S4). When the data collected in 2014 were compared to the 2012 data, oak mortality had increased significantly (Table S4). For instance, in the plot that was worst-hit by 2012, the % of dead oaks increased from 43.4% in 2012 to 50% in 2014, while in the least-hit plot by 2012, the percentage of dead oaks increased from 4.9% in 2012 to 8.2% in 2014. The greatest measured plot-level change in oak mortality was a jump to 26.3% in 2014 from 5.3% in 2012. The 2014 results show that oak mortality can progressively increase dramatically on a yearly basis after a year with rainfall substantially over the 30-year average, such as 2011. Mortality increased at different rates among plots, but in at least one plot it quintupled in just two years.

ANOVA determined that aridity as measured by the AI was different (*p* = 0.03) when comparing AI values of six plots from the peak season of 2009 (mean AI = 0.64, SE = 0.22), 2010 (mean AI = 0.71, SE = 0.12), and 2011 (AI = 1.46, SE = 0.28). Tukey’s test determined that the 2011 AI value, when widespread new oak infections occurred, was higher than that of 2009, while the 2010 was intermediate between the two. No new oak infection occurred in the dry 2009 and minimal new oak infection occurred in 2010, which was characterized by intermediate rainfall.

When we compared the seven hotspot sites containing four or more putative superspreader trees to the eight “cold spot” sites that contained no or few superspreaders, hotspots were characterized by higher bay laurel density and basal area (Table 2). While hotspots exhibited higher culturable and total (by culture and PCR) incidence of the pathogen in the wettest sampling period, and higher incidence of the pathogen by culturing during the driest sampling period (Table 2), model results show that these differences in incidence are driven by bay density rather than hotspot status per se (Table S5; Figure S1).

## 3. Discussion

Sudden Oak Death is a highly destructive introduced forest disease with a relatively young history in California [35] and characterized by different mechanisms of spread depending on the forest ecosystem affected [18]. Consequently, the relatively limited understanding of its epidemiology [20] has hampered the development and implementation of effective disease management approaches [13]. Although the literature on the topic is growing and providing an increasing body of information on aspects of the disease such as progression of tree mortality and its correlation with forest stand metrics [26,36,37], no study has yet explicitly tackled temporal and landscape-level patterns of infection on California bay laurel and coast live oak, nor identified drivers and spatial-temporal patterns of oak mortality. In coastal California’s mixed hardwood forests, California bay laurel has previously been identified as the major transmissive host for SOD, and the impacts of bay laurel infection on coast live oak have been well documented [18,21,22]. Hence, understanding patterns and drivers of heterogeneity in bay laurel or oak infection and in mortality of dead-end oak hosts, as well as uncovering patterns of bay-to-bay and bay-to-oak disease transmission, is critical.

Results of this study confirmed the importance of seasonal rainfall shifts in driving the prevalence of active infection in bay laurel hosts [29]. Active infection levels were highest in the spring and lowest in the autumn, corresponding to wet and dry periods, respectively, in California’s Mediterranean climate; this pattern holds true regardless of year and is confirmed by the logistic regression analysis between bay laurel infection and aridity index values. Furthermore, disease prevalence was higher in each seasonal survey in years with higher overall rainfall (Figure 1). The probability of bay laurel infection was affected by plot-level heterogeneity: infection was higher on east-facing slopes and increased with high bay density and low bay basal area, regardless of year (Figure 4A,B). In other words, dense stands of smaller bay trees on east-facing slopes had higher infection rates and were more likely to be hotspots that maintain active infection over most of the peak sampling periods, as well as contain refugial trees acting as reservoirs of infection in the dry late season or during droughts. 

Three plots had a sufficient number of superspreaders and refugial bay laurel trees to draw a comparison between the two categories. All refugial trees were found to also be superspreaders, however, the number of refugial superspreaders was almost an order of magnitude lower than the total number of superspreaders. This finding has important implications for control strategies and for future survey strategies: any future survey interested in identifying refugial trees will require an intensive survey design, given their low frequency. 

Because oaks, and not bay laurels, suffer high mortality in SOD outbreaks, understanding the factors that most strongly contribute to oak infection and mortality is critical. Oak infection was explained by oak DBH and bay density, as well as by proximity to the closest infected bay laurel tree. This proximity effect was strongest when oaks were between 0–5 m from an infected bay laurel; after 5 m, the likelihood of infection began to lessen, approaching zero after 10 m (Figure 4D). Oak mortality increased with the presence of cankers and decreased with increasing oak size; smaller cankered oaks had a higher risk of mortality than large oaks, despite large oaks becoming infected more easily (Figure 4E). Trees with a DBH of less than 10 cm had a higher probability of dying regardless of infection status, but trees exhibiting a canker were roughly 60% more likely to die than uninfected trees, and all trees were less likely to die as they increased in size, up to 60 cm. This could be because smaller trees that do become infected die more quickly than larger ones within a five-year window; those smaller trees could take less time to girdle, or it could be that cankers expand more rapidly in smaller trees. Note that the relationship between tree size and mortality for oaks with diameters larger than 60 cm was not clear because there were too few in the study to draw statistically-based conclusions; however many oaks with diameters over 60 cm have been killed by SOD since the beginning of the outbreak [38].

These results suggest several avenues for control efforts. Dense stands of younger bay laurel trees, especially those situated on east-facing slopes, should be prioritized for survey and possibly prophylaxis including stand thinning. A 10 m no-bay buffer zone around oak hosts may aid in preventing infection of specific oaks, as shown by Garbelotto et al. [26] based on measurements of airborne inoculum; however, it should be noted that larger oaks may require larger buffers than those required by smaller oaks. The relationship between likelihood of oak infection, distance from bay laurel, and oak diameter identified in this study can be used to determine a safe size for an effective “no-bay buffer” around target oaks or oak stands using the model presented in Figure 4D. Note that, as shown by Figure 4D, an effective “no-bay buffer” zone around oaks is less than 5 m for smaller oaks. However, buffers may not be effective if cryptic infection predated the creation of the buffer [16] or in cases where *P. ramorum* is spread by non-bay laurel hosts. In regard to the latter, however, it has been determined experimentally that oak infection requires very high inoculum pressure [26], so any host that may be relevant to oak infection must support very high levels of sporulation.

Bay disease incidence increased progressively each year between 2009 and 2011, due to progressively increasing levels of rainfall with each coming year (Figure 4F). However, except for a small number of newly infected oaks detected in 2011, no new oak infection was recorded until the 2012 survey. This pattern shows that: oak infection is not a yearly event, but only occurs when rainfall levels are above a certain threshold, presumably reached in this study during the wet season of 2010 and especially in the even wetter season of 2011, when rainfall was much greater than the 30-year average. Additionally, in 2011, the aridity index calculated in the late spring was significantly higher than the values calculated for the late springs of 2009 and 2010, indicating oak infection occurred during very wet conditions. This field result corroborates results of previous research in which inoculum levels necessary to obtain successful infection in experimental oak orchards were shown to be very high and occurred in nature only during exceptionally wet years [26]. Models predicting SOD-induced oak mortality must consider the clear presence of this threshold effect, and not simply correlate rainfall levels with oak infection levels; however, a simple positive correlation between rainfall and infection is supported by this study for bay laurel, a result that may also apply to other foliar host species. 

We note that new symptoms of infection in oaks became visible and were detected in only two sites in 2011 (Table S4), i.e., the year following the wet spring of 2010. In contrast, in 2012—a year after the very wet spring of 2011—new symptoms were detected across most of the entire plot network (Table S4, Figure 4F). These results further suggest that, in general, at least one year is necessary to consistently observe symptoms of natural SOD infection on oaks. 

Based on this study, the onset of oak mortality and of visible symptoms on living oak trees gradually increases over time since infection. Although, as stated above, symptoms become first visible 1 year after infection (based on the 2010–2012 results), oak mortality continues for at least 5 years after an infection year. This 5-year estimate is based on the likely assumption that oaks that were symptomatic early in the study had been infected in 2006, when rainfall levels were 139% of the 30-year average and very comparable to those of 2011, recorded as being 119% of the 30-year average. To further support such hypothesis, we note that novel oak infection was exceedingly high in 2006 all across coastal forests of the greater San Francisco Bay Area (Garbelotto, personal communication) and that multi-year data showed a decrease in asymptomatic oaks in 2006 [37]. Thus, oak infection and oak mortality, although inextricably interconnected, do not have matching time frames: oak infection occurs occasionally and in a relatively short window of time (e.g., within the spring of a very wet year), but the mortality that results will be staggered for at least 5 years after infection (Figure 4F). In 2011, most of the precipitation occurred in the spring, when temperatures were warmer, infection and thus sporulation were highest [26] and when oaks are most susceptible [39]. We suggest it is the combination of warm temperatures coupled with high levels of rainfall and high levels of inoculum co-occurring in the late spring that result in outbreaks of oak infection in coastal California forests as suggested by Garbelotto et al. [26]. In other words, rainfall during the winter may not necessarily lead to oak infection, unless temperatures are warm and approaching the 20 °C threshold shown to be ideal for infection of both bays and oaks [26,29].

Two important types of individual heterogeneity were observed in this study: individuals that persistently maintain active infection across seasons and years (superspreaders) and individuals that maintain active infection during suboptimal environmental conditions (refugial hosts). Although the design of the present study does not allow for tracking specific transmission events—and therefore, the actual effect of presumed superspreaders—it is reasonable to infer that, all other factors being equal, host trees harboring active infection more often will contribute more to overall infection levels. Conversely, refugial hosts may have an important effect on future disease cycles by starting new outbreaks at the onset of the wet season. It is also possible that locations harboring a larger number of putative superspreaders and refugial trees—i.e., disease hotspots—may be particularly important in driving epidemics. The results of the present study suggest that hotspot status does not significantly affect the cumulative disease incidence within a plot when bay density is accounted for. This result holds for both active and total infection assessed over the full study duration, and for total infection during the driest period. Thus, we conclude that in dense stand, reducing bay density is the most appropriate management strategy if the final goal is to reduce overall disease transmission rates. However, the same may not be true for less dense stands, where superspreaders and refugial hosts may play a more critical epidemiological role that needs to be further researched employing population genetics approaches. If superspreaders and refugial hosts in less dense stands are found to impact disease transmission in ways not measured in the present study, then focusing on these two host classes may be more cost effective than randomly removing bay laurels to reduce stand density. Regardless, removal of bays around specific oaks will lower the likelihood that those specific oaks will be infected.

In summary, this study has identified not only those site and weather variables associated with high infection levels by *Phytophthora ramorum*, both on California bay laurel and coast live oak, but also those variables that explain transmission from bay laurel to oak. Additionally, due to the repeated sampling over time, the study has identified potential superspreader trees that remain infectious for longer periods of time, as well as refugial trees that act as a reservoir of inoculum during dry periods. The study has also identified disease hotspots in which superspreaders and refugial trees were present in significant numbers. The information provided here is important to help further the understanding of the life cycle of the pathogen and the epidemiology of the disease and is relevant for the formulation of cost-effective and scientifically sound disease control options. While removal of the pathogen from the landscape is not possible [40], targeted management and knowledge of transmission patterns and disease cycles can help to curb its spread at the landscape level.

## 4. Materials and Methods

### 4.1. Site Selection and Experimental Design

This study was conducted within the San Francisco Public Utility Commission (SFPUC) peninsula watershed (37°31′10.3″ N 122°22′08.2″ W) in central San Mateo County, California. This site encompasses 9300 hectares of uneven-aged forest with some oaks surpassing 100 years of age, ranges in elevation between 95 and 1050 m, and hosts a variety of habitats, including riparian, coastal heathlands, redwood-tanoak stands, and mixed-oak woodlands. In the study plots, coast live oaks and bay laurels were dominant and together represented over 80% of the tree population: other tree species present in the plots were tanoaks, redwoods (*Sequoia sempervirens*), Douglas-firs (*Pseudotsuga menziesii*), Pacific madrones (*Arbutus menziesii*), and Canyon live oaks (*Quercus chrysolepis*). Douglas-firs and redwoods are foliar hosts with limited sporulation potential and a very small epidemiological role [28]. Pacific madrones are foliar and branch hosts with some sporulation potential but with an unknown epidemiological role [41]. Canyon live oaks, instead, are both stem and branch hosts but support minimal sporulation and have no epidemiological role in the spread of SOD. The watershed has maintained limited public access for nearly a century, thus reducing the amount of anthropogenic disturbance. Mean annual temperatures range from 8.8 to 21.5 °C, with the coldest temperatures occurring in January and the warmest in September. The mean annual precipitation is 62 cm, with the majority of rainfall occurring from November to April [33]. This site was likely to have been infested by *P. ramorum* 10–20 years prior to plot establishment, with confirmed reports of *P. ramorum* dating as early as 2001 [42]. Temperature data were collected from two remote automated weather stations (RAWS): Pulgas (latitude: 37.47500, longitude: −122.29810) in the Crystal Springs drainage, and Spring Valley (latitude: 37.5625, longitude: −122.436389) in the Pilarcitos drainage. Microclimate data were collected via three HOBO data loggers (Onset Computer Corporation, Bourne, MA, USA) in six study sites. Rainfall data were retrieved from the California Department of Water Resources (https://water.ca.gov last accessed on 1 October 2021), from the SFPUC data archives of the Lower Crystal Springs rain gauge (latitude: 37.5325, longitude: −122.363) and from the Pilarcitos rain gauge (latitude: 37.547, longitude: −122.421). Rainfall data from neighboring San Francisco were obtained at https://ggweather.com/sf (accessed on 25 January 2022). Using temperature and rainfall values, an aridity index (AI) was calculated of each of six plots as AI = 12 × P/(T + 10) for 2009–2011, where P is the monthly precipitation in mm and T is the mean monthly air temperature in degrees °C [43]. A high index value represents a low level of aridity, whereas a low index indicates high aridity.

In the summer of 2008, 16 research plots within the watershed were established in locations where suitable plant hosts were present; however, only 15 sites contained both oaks and bay laurels, were originally infested by SOD, and thus were included in the study (Table S1). The plots were spaced at least 2 km apart to minimize potential spatial autocorrelation interference between sites. This distance was based on results of previous population genetics studies and spatial autocorrelation analyses on the mobility of infectious propagules that suggested the vast majority of airborne inoculum moves mostly tens or hundreds of meters, while only exceptionally that distance can reach 1–2 km [44]. Three transects were laid out in each plot, while, in six plots (three per major drainage), the number of transects was doubled with the aim of obtaining more isolates of the pathogen for a concurrent population genetics study [45]. In total, there were 66 transects nested within the 16 research plots. Each transect was 100 m in length and 10 m in width, radiating from a plot center. All bay laurel and coast live oak stems with a minimum diameter at breast height (DBH) of 1 cm were tagged, and the DBH was recorded in cm. Any major branches of a tree separated from one another below breast height (1.4 m) were considered independent stems. At 10-m increments along each transect, a single bay laurel stem (when present) was selected for repeated surveying throughout the year, while all oak stems occurring within each 100 × 10 m transect corridor were inspected in the fall of each year for five years. 

### 4.2. Field Surveys

Following plot network establishment and preliminary data collection in the summer of 2008, bay laurel assessments were repeated 10 times over a four-year period spanning 2009–2012. Surveys were conducted three times per year in 2009–2011 during predetermined seasonal periods in order to capture snapshots of pathogen viability throughout the year. These seasonal periods were defined: an “early” season sampling in late winter/early spring (February/March) to capture pathogen dynamics when temperatures were not yet warm enough for maximum sporulation; a “peak” season sampling in late spring/early summer (May/June), when rainfall coupled with warmer temperatures provide ideal conditions for sporulation and infection [29]; and a “late” season sampling in late summer/early autumn (September/October) after the hottest and driest months have typically urged the pathogen into dormancy [33,45]. A single end-point survey took place in the late spring, or peak season of 2012. An additional late season survey of all plots was performed in the fall of 2014 using identical field and laboratory methodologies. This last dataset is not included in the analyses, but it is presented in the Supplementary Materials and is briefly discussed.

Bay laurel analyses consisted of surveying a total of 388 stems that occurred at 10 m intervals along each transect. If a tree was determined to be symptomatic following an initial visual assessment, six symptomatic leaves were then sampled from the lower canopy and tested for presence of the pathogen via culturing and qPCR. Symptomatic leaves were recognized by areas of tissue necrosis often congregated at the tip, or along the lower edge of a leaf where droplets of water congregate and remain. These lesions are classically characterized by a yellow halo just beyond an active zone of infection that appears as a dark irregularly shaped line, or as dark pixilated spots scattered about the leaf, more common of an active infection [21,23]. Only leaves from trees with visible symptomatic tissue were collected and processed, because, in the absence of visible symptoms, a positive ID via molecular diagnostics is unlikely [46]. A total of 3880 data points (from 388 trees) were obtained for determining bay laurel infection across the 10 sampling events. 

An initial total of 950 oaks across all transects were surveyed once per year, during the autumn or late sampling, over a five-year period spanning 2008–2012. An additional oak survey and sampling occurred in the fall of 2014: results of that sampling are not included in the analyses, but they are presented in the Supplementary Materials and briefly discussed. Oak tissue was sampled only for those trees that exhibited the bleeding cankers that are a visible sign of infection [23,27,38]. Cankers were sampled by removing the outer layer of bark, excising the margin of infected cambial tissue with a sterile scalpel and embedding chips from the margins of each canker directly into PARP selective medium [27]. A positive pathogen identification required growth of a colony that could be morphologically identified as *P. ramorum* [47]. Infected stems were also surveyed for the presence of bark beetles and the secondary sapwood fungus *Annulohypoxylon thouarsianum* as indicators of incipient mortality, and the approximate level of canopy dieback for each tree was noted. At each surveyed oak, the distance to the nearest infected bay laurel foliage was measured in meters in a straight line from the trunk of the oak to the leaves of the bay laurel branch closest to the oak. Canopy cover was assessed at each surveyed tree using a spherical densitometer comprised of 24 squares, held at waist height 12–18 inches from the body. Four readings were taken, one for each cardinal direction using the following method: four dots spaced evenly across each of the 24 squares were assumed and the number of dots not covered by the reflecting canopy were tallied, averaged, multiplied by 1.04 and subtracted from 100, resulting in the percent of canopy coverage [48]. 

The slope and aspect at each oak and bay laurel tree were measured using a Suunto MC-2 compass with clinometer. Geographic location and elevation were recorded using a Garmin GPSMAP 60Cx. Bay laurel basal area and density were calculated for each 100 × 10 m transect corridor and site level data was obtained by averaging the corridors’ data and scaling the average value to a per hectare measurement. Basal area of each individual bay tree within a transect was calculated using the following equation (BA = π × (DBH in cm × 0.01 × 0.5)^2^) and then summed to determine the basal area per transect. Results from multiple transects per plot were averaged and then scaled to a per hectare measurement. Circular aspect variables were transformed to the linear variables of northness = cos (aspect) and eastness = sin (aspect) (Roberts, 1986) where values range from −1 to 1, and all additional variables used in the models were centered and scaled (subtracting from the mean and dividing by the standard deviation) prior to analysis. Stems missing data for any variable were excluded from the analyses. Density was calculated by counting all the stems above 1 cm DBH that were in each transect corridor; results from multiple transect corridors were averaged and then scaled to a per hectare measurement.

### 4.3. Pathogen Isolation and In Vitro Culturing

Within 72 h of sampling, each collected leaf had a small section of symptomatic tissue excised along the advancing margin of a single lesion and embedded in the *Phytophthora*-selective medium PARP [27]. Plates were then incubated in the dark at 20 °C for 7–10 days. All plates exhibiting mycelial growth were examined using microscopy and morphologically determined to be *P. ramorum* positive or negative [47]. Oak canker samples plated directly onto PARP in the field were incubated for 3–5 days and then subcultured to reduce contaminants before colonies were identified as *P. ramorum* based on morphology.

### 4.4. Molecular Diagnostics

Given that the foliar symptoms caused by *P. ramorum* are rather generic, symptomatic leaf samples that were culture-negative for *P. ramorum* could have been infected by another species of *Phytophthora* [33]. Alternatively, infection may have been present, but *P. ramorum* may have entered dormancy or may have died, becoming unculturable. In order to determine whether a *P. ramorum* culture-negative was a false negative (i.e., *P. ramorum* was present but unculturable) or a true negative (*P. ramorum* was truly absent), a molecular PCR diagnostic assay highly specific for *P. ramorum* was conducted on all symptomatic bay laurel leaves that did not yield a positive culture. A surface-sterilized 6 mm hole punch was used to excise leaf sections from the margin of putative *P. ramorum* lesions. From each set of six leaves, three leaf punches were selected and combined in a 2 mL screw-top tube containing a sterile glass bead. Samples were lyophilized, then pulverized using a FastPrep-24™ homogenizer (MP Biomedicals, Irvine, CA, USA) for a minimum of 30 s at 4 rmps. DNA was extracted using either the CTAB and phenol/chloroform extraction protocol of Hayden et al. [49], or the ROSE extraction method of Steiner et al. [50] as modified by Osmundson et al. [51]. Presence of *P. ramorum* in culture-negative leaves was assessed using the nested qPCR assay described by Hayden et al. [46]. In order to increase the sensitivity of the assay, a nested protocol is preferred, as evidenced by the fact that the first PCR protocol to be officially approved by the USDA for the official detection of *P. ramorum*, a highly regulated plant pathogen [35], was in fact a nested protocol [49]. PCR reactions were performed on a Bio-Rad (Hercules, CA, USA) CFX96 Touch Real Time PCR Detection machine.

### 4.5. Statistical Analyses

Analyses were divided into the following three categories and run in several separate models. The first category focused on factors explaining infection of coast live oak and/or bay laurel by *P. ramorum*, the second was focused on factors explaining the survival potential of infected oaks, and the third was run to determine the association between cumulative disease incidence (CDI), i.e., the total proportion of individuals infected at any point during the study period—and specific plot-level characteristics. All statistical analyses were conducted in the R statistical environment, version 3.1.0 [52]. Generalized linear mixed effects models (GLMM) were constructed using the lme4 package [53]. Akaike’s Information Criterion (AIC) was used to select the models that best fit the data. Several variables were used in the models as predictors, including topographic characteristics (slope, aspect), the biotic neighborhood (canopy cover, bay density, bay basal area of the transect) and individual descriptors (DBH, proximity to nearest bay laurel). Models with and without interaction terms were compared using F-tests; as differences between these models were not found to be statistically significant, interaction terms were omitted from the models reported here. 

In the first set of analyses, the likelihood of host infection was calculated using two separate models, one for bay laurel and one for coast live oak. The likelihood of active infection occurring on bay laurel was examined using a repeated measures GLMM to investigate how neighborhood effects and temporal variation may be associated with *P. ramorum* prevalence on bay laurel. Active bay laurel infection was the dependent variable, with each tree scored for having a viable *P. ramorum* infection confirmed through culture methods. The model included the independent predictor variables of bay laurel DBH (bayDBH), bay laurel density (bayDens), bay laurel basal area (bayBA), aspect (northness vs. eastness), and canopy cover with sampling period (season = early, peak, or late), and year of sampling (2009–2012) as fixed effects. The random effect variable of bay laurel stem ID (TAG) accounted for the repeated measure of stems through time. To account for spatial non-independence of stems located within the same plot or along the same transect, the random variables of transect nested within plot were included. The intercept of the model was allowed to shift/vary among groups denoted by the random variables. The model is summarized by the following formula: (ActiveBayLaurelInfection ~ t.bayDBH + t.bayBA + t.bayDens + northness + eastness + CanopyCover + t.Season + t.Year + (1 | TAG) + (1 | PlotID/TransectID)), where “t.” indicates data were scaled and transformed as previously described. Although rainfall was not included in the models above, due to the fact that data were not available for each plot, a logistic regression was used to determine the presence of a correlation between the percentage of infected bay laurels, using infection determined by combined culturing and PCR data, and the aridity index AI, using rainfall and temperature data we had collected from six plots (Tables S2 and S3).

Likelihood of oak infection (confirmed via cultured isolates from symptomatic canker tissue) was determined with a model similar to the bay laurel infection model outlined above, but excluding the sampling period term, as oaks were only sampled only once per year. The fixed predictor variables of oak DBH (oakDBH) and distance (m) to the nearest infected bay laurel foliage (prox) were added to the model. The model employed to estimate likelihood of oak infection is summarized by the following formula: [ActiveOakInfection ~ t.oakDBH + t.bayBA + t.bayDens + t.prox + eastness + northness + (1 | PlotID/TransectID). In order to determine whether new oak infections observed in 2010, and especially in 2011 (Table S4), were truly associated with decreased aridity driven by increased rainfall, the AI values calculated for six representative plots in the late spring or peak sampling of 2010 and 2011 (Table S3) were compared with those measured in the late spring of 2009 through an ANOVA, and homogeneous groups were identified using Tukey’s test.

With regard to the second category of analyses, a third model was run in order to examine the survival of infected oaks over the course of the study. Oak mortality at each sampling point was used as the dependent variable in a repeated measures logistic regression GLMM, identical to the models described above, but in this case the presence of a visible canker was added as a fixed predictor variable to the model, as shown by the following formula: (OakSurvival ~ t.oakDBH + t.bayBA + t.bayDens + t.prox + eastness + northness + cankered + (1 | PlotID/TransectID)).

The third category of analyses included an additional set of models run to determine the association between cumulative disease incidence (CDI)—the total proportion of individuals infected at any point during the study period—and plot-level characteristics including bay laurel density, bay laurel basal area, and whether or not the plot is a disease “hotspot.” We defined a hotspot as a plot containing four or more putative “superspreaders,” here defined as trees that yielded active (culture-positive) infections in 7 or more of the 10 sampling events (i.e., ≥70% individual active infection frequency). This threshold was chosen because 7/10 culture positives would mean a tree was infectious in all of the early and peak season sampling events and/or at least one of the late season sampling events. Generalized linear models examined CDI for both culture-positive and total (culture-positive and culture-negative but PCR-positive) infection for the wettest (peak 2011), driest (late 2009), and all sampling periods, as a function of the explanatory variables bay density, bay basal area, and “hotspot” status. Models incorporated quasibinomial errors due to overdispersion. Though not incorporated into the models, hotspot and non-hotspot plots were also assessed for the presence of putative refugial hosts, here defined as individual trees in which active infection was observed in two or more of the drier late season sampling events. 

In order to identify site or disease characteristics that may be correlated with the presence of disease superspreaders and of refugial trees, we compared the following metrics for sites containing superspreaders and refugial trees (aka hotspots) to those for sites containing few or no superspreaders (aka cold spots): tree density, basal area, disease incidence (DI) in the spring of the year when infection was highest during the entire study (2011) and in the Fall of the year when infection was lowest (2009). Disease incidence was analyzed separately for pooled positive values (obtained by adding trees that were culture positive for *Phytophthora ramorum* to trees that were PCR positive for *P. ramorum*) and for values obtained only considering culture positives. Continuous numerical variables, such as density and basal areas were compared using Student’s *t*-test, while disease incidence values, expressed as the proportions of a sample positive for the disease, were compared using two-tailed Fisher’s exact tests. 

## Figures and Tables

**Table 1:** Models listing the effect of several variables on the likelihood of infection of bay laurel (*Umbellularia californica*) and oak (*Quercus agrifolia*) hosts, as well as the survival of infected oak hosts. Bold text indicates a significant result. Significant results with a negative sign denote lower infection or survival likelihood with increases in that variable. Results with a positive sign denote higher infection or survival likelihood with increases in that variable. In the case of season, “Season Early” and “Season Late” are compared to “Season Peak”, when the highest bay laurel infetion levels were recorded.

	INFECTION PREDICTION												SURVIVAL PREDICTION					
Group	*U. californica*						*Q. agrifolia*						*Q. agrifolia*					
RANDOM EFFECTS	Variance			(Std. Dev.)			Variance			(Std. Dev.)			Variance			(Std. Dev.)		
Tree ID	0.4499			0.6707														
Transect (Plot)	0.3325			0.5766			0.8221			0.9067			0.0000			0.0000		
Plot	0.8729			0.9343			0.2211			0.4702			1.1172			0.3424		
**FIXED EFFECTS**	**Estimate**		**(SE)**		***p* Value**		**Estimate**		**(SE)**		***p* Value**		**Estimate**		**(SE)**		***p* Value**	
(Intercept)	**1.05**		**(0.40)**		**0.009**													
Bay Laurel DBH	−0.08		(0.07)		0.235													
Bay Basal Area	**−0.31**		**(0.14)**		**0.031**		−0.03		(0.33)		0.923		0.05		(0.22)		0.83	
Bay Density	**0.44**		**(0.17)**		**0.010**		**0.83**		**(0.35)**		**0.017**		0.09		(0.23)		0.71	
Northness	0.09		(0.08)		0.303		−0.19		(0.17)		0.250		−0.22		(0.17)		0.19	
Eastness	**0.20**		**(0.09)**		**0.022**		−0.17		(0.16)		0.311		0.05		(0.17)		0.78	
Canopy Cover	−0.003		(0.003)		0.347													
Oak DBH							**0.94**		**(0.15)**		**4.41 × 10^−10^**		**−0.29**		**(0.14)**		**0.04**	
Proximity to Infected Bay							**−1.2**		**(0.15)**		**1.81 × 10^−15^**		−0.10		(0.16)		0.511	
Cankered													**3.027**		**(0.30)**		**<2 × 10^−16^**	
Season Peak																		
Season Early	**−1.21**		**(0.12)**		**<2 × 10^−16^**													
Season Late	**−1.38**		**(0.12)**		**<2 × 10^−16^**													
Year 2009	**−3.18**		**(0.14)**		**<2 × 10^−16^**													
Year 2010	**−1.48**		**(0.11)**		**<2 × 10^−16^**													
Year 2011	**−0.57**		**(0.15)**		**0.0002**													
**Model AIC**	3434						617.5						518.4					

**Table 2:** Comparisons of four metrics between hotspots and cold spots. For a definition of hotspots and cold spots see the text. Bay laurel density and basal areas were compared using *t*-tests, while cumulative and single time disease indices (DI) were compared using 2-tailed Fisher exact tests. Fall of 2009 and spring of 2011 were the driest and wettest sampling times of the entire study, respectively.

Metric	Plot Type		*p* Value
	Hot Spots (7 Plots)	Cold Spots (8 Plots)	
Average bay laurel density (number of trees/ha), SE	126.47, 47.8	70.83, 14.5	0.04
Average bay laurel basal area (square m/ha), SE	11.4, 2	5.54, 1.7	0.05
CDI * by PCR and culturing, % positive (sample size)	61.5% (2540)	41.57% (1340)	0.0001
CDI by culturing only, % positive (sample size)	40% (2540)	18.43% (1340)	0.0001
Fall 2009 DI by PCR and culturing (% positive of sample size)	47.64% (254)	44.03% (134)	0.52
Fall 2009 DI by culturing only (% positive of sample size)	8.27% (254)	1.49% (134)	0.0059
Spring 2011 DI by PCR and culturing (% positive of sample size)	88.19% (254)	70.15% (134)	0.0001
Spring 2011 DI by culturing only (% positive of sample size)	77.17% (254)	54.48% (134)	0.0001

* CDI = cumulative disease incidence across all sampling times.

## Data Availability

Not applicable.

## Supplementary Materials

The following supporting information can be downloaded at: https://www.mdpi.com/article/10.3390/pathogens11020250/s1, Figure S1: proportion of individuals infected within sampling plots with respect to bay density and plot hotspot status; Table S1: location, elevation and bay laurel density of sampling plots; Table S2: percentages of California bay laurel trees that were positives for *Phytophthora ramorum* by culturing and PCR by plot and sampling time; Table S3: values of the Aridity Index calculated in six study sites for the 30 day-period before sampling times; Table S4: percentages of living and dead coast live oaks that were positive for *Phytophthora ramorum* by culturing in different study sites and at different sampling times; Table S5: models assessing plot-level disease incidence (proportion of infected individuals) with respect to bay density and plot hotspot status.
