# Supplementary material for: The Epidemiology of Sudden Oak Death Disease Caused by Phytophthora ramorum in a Mixed Bay Laurel-Oak Woodland Provides Important Clues for Disease Management
Source: Pathogens. 2022 Feb 15;11(2):250. doi: 10.3390/pathogens11020250 (PMC8874385; doi:10.3390/pathogens11020250)
Supplement: Supplementary file 1 [file pathogens-11-00250-s001.zip › pathogens-1551562-supplementary.pdf]

Supporting Table S1.
Locations of sampling plots located within the SFPUC Peninsula watershed and associated plot data including geographical coordinates, elevation, and density of California bay laurel (*Umbellularia californica*) trees.

Drainage/Area	Plot	Latitude	Longitude	Elevation (m)	Bay Density (# /0.33ha)
CS	0	37.53420725	-122.3765705	115	59
CS	1	37.47667587	-122.3240183	131	8
CS	2	37.57758904	-122.4122678	155	56
CS	3	37.48812832	-122.3420616	107	39
NA	5	37.45370812	-122.3306575	457	2
CS	6	37.54543221	-122.4033964	104	113
CS	7	37.46138871	-122.3038186	140	25
CS	8	37.55632374	-122.3955187	113	299
PL	9	37.53828797	-122.4080535	252	70
CS	10	37.56582756	-122.4038403	187	42
PL	11	37.53001152	-122.3908418	295	27
CS	12	37.51191507	-122.3585746	95	51
PL	14	37.54701261	-122.4284439	311	39
PL	15	37.52467467	-122.3933726	197	70
CS	16	37.55031173	-122.3838935	129	33

CS=Crystal Springs drainage; PL=Pilarcitos drainage; NA=Not associated with specific drainage.

Supporting Table S2.

Percentages of California bay laurel trees that were positives for *Phytophthora ramorum* by culturing and PCR by plot and sampling time. Data from plots 0, 8, 9, 12, 15 and 15 were used in logistic regression analysis with Aridity Index values presented in Table S3.

		EARLY 2009		PEAK 2009		LATE 2009		EARLY 2010		PEAK 2010		LATE 2010		EARLY 2011		PEAK 2011		LATE 2011		PEAK 2012	LATE 2014	
Plot ID	# Surveyed	% Culture+	% *P.ramorum* +	% Culture+	% *P.ramorum* +	% Culture+	% *P.ramorum* +	% Culture+	% *P.ramorum* +	% Culture+	% *P.ramorum* +	% Culture+	% *P.ramorum* +	% Culture+	% *P.ramorum* +	% Culture+	% *P.ramorum* +	% Culture+	% *P.ramorum* +	% Culture+	% Culture+	% *P.ramorum* +
0	28	3.6	21.4	0	42.9	0	46.4	3.6	39.3	7.1	39.3	0	7.1	46.4	64.3	71.4	78.6	25	75	39.3	14.8	22.2
1	8	0	37.5	0	12.5	0	62.5	0	12.5	0	12.5	0	0	0	0	62.5	75	25	25	62.5	33.3	50
2	30	3.3	16.7	23.3	60	3.3	46.7	30	70	63.3	70	53.3	63.3	80	96.7	83.3	96.7	56.7	96.7	43.3	23.3	26.7
3	16	0	0	0	31.3	0	25	6.3	31.3	0	18.8	0	12.5	37.5	43.8	81.3	81.3	62.5	81.3	62.5	21.4	21.4
5	2	0	0	0	0	0	50	0	0	0	0	0	0	0	0	0	0	0	0	50	50	50
6	21	23.8	52.4	57.1	85.7	23.8	81	76.2	100	90.5	95.2	47.6	76.2	90.5	100	95.2	100	61.9	100	47.6	63.6	72.2
7	19	10.5	31.6	15.8	36.8	0	31.6	10.5	26.3	21.1	36.8	15.8	15.8	26.3	36.8	36.8	47.4	10.5	26.3	31.6	5.3	21
8	66	22.7	50	31.8	86.4	13.6	69.7	22.7	63.6	69.7	87.9	48.5	62.1	86.4	89.4	80.3	92.4	59.1	93.9	39.4	30	53.3
9	45	0	15.6	13.3	28.9	0	22.2	11.1	28.9	42.2	53.3	2.2	20	48.9	68.9	66.7	82.2	24.4	60	91.1	24.4	34.1
10	28	10.7	57.1	21.4	78.6	17.9	78.6	39.3	75	57.1	89.3	57.1	60.7	82.1	92.9	96.4	100	78.6	96.4	78.6	7.4	44.4
11	13	0	7.7	0	7.7	0	46.2	0	15.4	23.1	30.8	30.8	38.5	30.8	53.8	38.5	53.8	0	0	53.8	7.7	7.7
12	24	16.7	41.7	8.3	45.8	8.3	54.2	8.3	45.8	12.5	54.2	4.2	8.3	4.2	41.7	25	54.2	12.5	33.3	45.8	37.5	54.2
14	28	0	0	3.6	17.9	0	17.9	21.4	57.1	35.7	67.9	28.6	39.3	28.6	53.6	42.9	71.4	0	39.3	32.1	15.4	26.9
15	45	0	2.2	4.4	17.8	2.2	13.3	6.7	22.2	33.3	40	8.9	20	62.2	84.4	75.6	86.7	40	73.3	86.7	18.6	41.9
16	15	0	40	0	66.7	0	80	0	13.3	20	73.3	6.7	13.3	46.7	66.7	80	86.7	6.7	80	53.3	0	20

Supporting Table S3.

Aridity Index values in six study sites calculated for the 30 day-period before sampling times.

Plot	Early 2009	Peak 2009	Late 2009	Early 2010	Peak 2010	Late 2010	Early 2011	Peak 2011	Late 2011
**0**	0.52651795	0.27464789	0	2.43421658	0.73344117	0	4.23897582	0.7020339	0
**8**	5.17389886	0.01033295	0.0654809	1.88161926	0.73636812	0	4.13483146	0.84438201	0
**12**	4.85900358	0.27745665	0.06481439	2.63041905	0.80778114	0	2.99544304	0.93481378	0
**9**	8.81927711	1.24247788	0.10261108	4.59932168	0.9357713	0.00934874	5.13752092	2.05941835	1.19646505
**14**	8.81927711	0.80264672	0.09772449	4.67461313	0.12450577	1.42571757	5.71910112	2.14062333	0.02776281
**15**	8.81927711	1.24247788	0	3.44358562	0.93700994	0.02900392	5.13602077	2.07264093	0.0291058

Supporting Table S4. Percentages of living and dead coast live oaks that were positive *for Phytophthora ramorum* by culturing in different study sites and at different sampling times.

		2009		2010		2011		2012		2014	
plot	# Live oaks	% Infected	% Dead	% Infected	% Dead	% Infected	% Dead	% Infected	% Dead	% Infected	% Dead
0	123	12.2	19.5	7.3	22.8	3.3	24.4	4.1	28.5	5.7	32.5
1	132	0.0	0.8	0.0	0.8	0.0	0.8	0.0	5.3	3	6.1
2	74	14.9	20.3	13.5	23.0	10.8	27.0	16.2	27.0	6.8	33.8
3	52	18.9	24.5	17.0	30.2	13.2	40.0	15.1	43.4	11.5	50
5	0	0.0	0.0	0.0	0.0	0.0	0.0	0.0	0.0	0	0
6*	35	0.0	0.0	0.0	0.0	0.0	0.0	2.8	0.0	17.1	8.6
7	86	12.8	0.0	12.8	0.0	1.2	10.4	1.2	10.4	11.6	16.3
8	34	23.5	29.4	17.6	32.4	14.7	35.3	23.5	38.2	23.5	44.1
9	31	9.7	16.1	6.5	22.6	6.5	25.8	9.7	25.8	12.9	12.9
10	106	13.2	13.2	8.5	17.9	4.7	19.8	6.6	19.8	15.1	25.5
11	61	4.9	1.6	3.3	4.9	3.3	4.9	3.3	4.9	1.6	8.2
12	123	12.2	5.7	7.3	8.1	6.5	12.2	7.3	13.8	15.4	9.8
14	37	0.0	2.6	0.0	2.6	10.5	5.3	13.2	10.5	10.8	24.3
15	19	0.0	0.0	0.0	5.3	5.3	5.3	15.8	5.3	5.3	26.3
16	72	8.2	1.4	6.8	4.1	6.8	5.5	6.8	5.5	16.7	6.9

* Canyon Live Oak

Supporting Table S5.
Models assessing plot-level disease incidence (proportion of infected individuals) with respect to bay density and plot hotspot status, defined as containing four or more superspreader individuals that harbored active (culture-positive) *P. ramorum* infection in ≥ 70% of sampling events.

All sampling periods, total infection

Coefficients:

	Estimate	Std. Error	t value	Pr(>|t|)	
(Intercept)	-0.817104	0.440676	-1.854	0.0907	.
bay_density	0.005762	0.002082	2.767	0.0183	*
bay_basal_area	0.020989	0.031197	0.673	0.5150	
statusnon-hotspot	-0.252599	0.350846	-0.720	0.4866	

All sampling periods, active (culture-positive) infection

Coefficients:

	Estimate	Std. Error	t value	Pr(>|t|)	
(Intercept)	-1.569650	0.272398	-5.762	0.000126	***
bay_density	0.003019	0.001011	2.986	0.012376	*
bay_basal_area	0.048596	0.019517	2.490	0.030039	*
statusnon-hotspot	-0.541517	0.222518	-2.434	0.033198	*

Wettest sampling period, total infection

Coefficients:

	Estimate	Std. Error	t value	Pr(>|t|)
(Intercept)	-0.672460	1.122963	-0.599	0.561
bay_density	0.015056	0.009706	1.551	0.149
bay_basal_area	0.045254	0.075260	0.601	0.560
statusnon-hotspot	-0.075388	0.814156	-0.093	0.928

Wettest sampling period, active (culture-positive) infection

Coefficients:

	Estimate	Std. Error	t value	Pr(>|t|)	
(Intercept)	-0.646582	0.879808	-0.735	0.478	
bay_density	0.008377	0.005507	1.521	0.156	
bay_basal_area	0.044188	0.060559	0.730	0.481	
statusnon-hotspot	-0.185739	0.672978	-0.276	0.788	

Driest sampling period, total infection

Coefficients:

	Estimate	Std. Error	t value	Pr(>|t|)	
(Intercept)	-0.902654	0.719325	-1.255	0.2355	
bay_density	0.007036	0.003233	2.176	0.0522	.
bay_basal_area	-0.041267	0.052131	-0.792	0.4453	
statusnon-hotspot	0.542877	0.587822	0.924	0.3755	

Driest sampling period, active (culture-positive) infection

Coefficients:

	Estimate	Std. Error	t value	Pr(>|t|)	
(Intercept)	-4.784164	1.650418	-2.899	0.0145	*
bay_density	0.005208	0.003985	1.307	0.2179	
bay_basal_area	0.095617	0.115601	0.827	0.4257	
statusnon-hotspot	-0.841571	1.458281	-0.577	0.5755	

Significance codes: ***0.001 **0.01 *0.

Supporting Figure S1.

Proportion of individuals infected within sampling plots with respect to bay density and plot hotspot status, defined as containing four or more superspreader individuals that harbored active (culture-positive) *P. ramorum* infection in $\geq$ 70% of sampling events. Lines show predicted values for a tree with the median value of bay basal area across all plots. Hotspot plots are shown in red, non-hotspot plots in blue. Colored background represents the 95% confidence interval. A. Total (culture-positive or PCR-positive) infection across the entire study period. B. Active (culture-positive only) infection across the entire study period. C. Total infection during the wettest sampling event (Peak 2011). D. Active infection during the wettest sampling event. E. Total infection during the driest sampling event (Late 2009). F. Active infection during the driest sampling event.

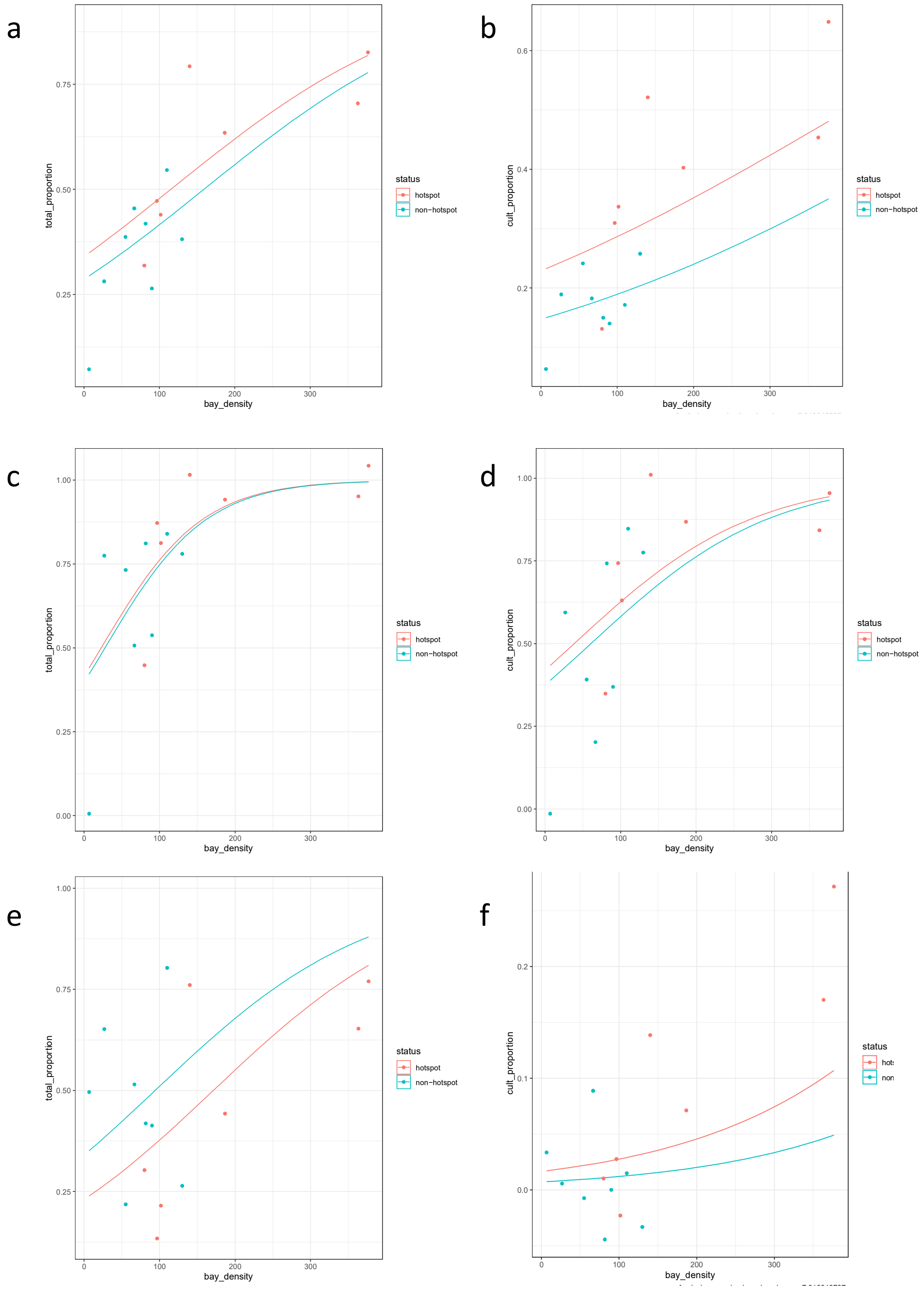
a
b
c
d
e
f
total_proportion
cult_proportion
bay_density
0
100
200
300
0.25
0.50
0.75
1.00
0.00
0.0
0.1
0.2
0.4
0.6
status
hotspot
non−hotspot
